# A phase II trial of post-operative chemoradiotherapy for completely resected gastric cancer with D2 lymphadenectomy

**DOI:** 10.3892/ol.2014.2382

**Published:** 2014-07-25

**Authors:** YU-JIE ZHAI, YI-PENG SU, SHENG-JIE WANG, FANG-LING NING, ZHEN-BO WANG, WEN-ZHENG YU, SHAO-SHUI CHEN

**Affiliations:** 1 Binzhou Medical University, Affiliated Hospital of Binzhou Medical University, Binzhou, Shandong, P.R. China; 2 Department of Oncology, Affiliated Hospital of Binzhou Medical University, Binzhou, Shandong, P.R. China; 3 Department of Hematology, Affiliated Hospital of Binzhou Medical University, Binzhou, Shandong, P.R. China

**Keywords:** gastric cancer, D2, chemoradiotherapy, post-operative

## Abstract

The optimal post-operative adjuvant treatment for completely resected gastric cancer with D2 lymphadenectomy remains controversial. The present study was a phase II trial on post-operative chemoradiotherapy in 30 patients with gastric cancer. Patients with stage II to IV (M0) gastric cancer received two cycles of chemotherapy prior to and following chemoradiotherapy. The chemotherapy consisted of a 2-h infusion of oxaliplatin (100 mg/m^2^) and folinic acid (100 mg/m^2^), which was followed by a 46-h continuous infusion of 5-fluorouracil (5-FU; 2,400 mg/m^2^) through a portable pump, repeated every 3 weeks. The chemoradiotherapy consisted of 45 Gy of radiotherapy for 5 weeks and 5-FU continuous infusion (350 mg/m^2^/day). In total, 30 patients were enrolled in this study. All patients underwent the chemoradiotherapy treatment as planned. A total of 10 (33.3%) patients relapsed; two (6.7%) locoregional relapses and mediastinum metastases, four (13.3%) peritoneal relapses, and four (13.3%) distant metastases. The three-year overall survival and disease-free survival rates were 72.7 and 65%, respectively. The toxicities of chemotherapy and radiotherapy, consisting of neutropenia, nausea and hand-foot syndrome, were observed. In conclusion, post-operative chemoradiotherapy following complete resection of gastric cancer with D2 lymphadenectomy is feasible in a significant subset of patients.

## Introduction

Gastric cancer is the fourth most common type of cancer and the second highest cause of cancer-related mortality worldwide (1). The incidence of gastric cancer varies widely among different regions globally, with almost two-thirds of all cases in developing countries and 42% in China alone (1). In spite of the progress in diagnostic techniques and the decline in the overall incidence of gastric cancer, the majority of patients still experience recurrence following a complete resection with negative margins. In addition, the majority of cases are diagnosed at an advanced stage. Therefore, gastric carcinoma remains a therapeutic challenge, as its treatment requires surgery and post-operative chemotherapy and/or radiotherapy (2).

Several studies have shown that adjuvant treatment enhances survival (3). Chemoradiotherapy has recently been associated with significantly enhanced survival times in gastric cancer; this was primarily discussed in Intergroup Study INT-0116, which is one of the chief studies of adjuvant trials published in 2001. The trial included 556 stage IB-IV (M0) gastric cancer patients following R0 surgery, 275 randomized cases in a control group and 281 cases in a chemoradiotherapy group (4). The results showed that post-operative chemoradiotherapy prolonged the three-year relapse-free survival rate and the three-year overall survival (OS) rate compared with surgery alone, with statistically significant differences (48 vs. 31%, P<0.001; and 52 vs. 41%, P=0.005, respectively). In 2012, according to the 10-year follow-up data for the INT-0116 study, it was found that the OS and relapse-free survival times were enhanced by chemoradiotherapy compared with the control group (35 vs. 27 months; HR, 1.32; P=0.0046; and 27 vs. 19 months; HR, 1.51; P<0.001) (5). Since the publication of the results from the INT-0116 study, adjuvant chemoradiotherapy has become the standard therapy following curative resection in North America for patients with gastric cancer. Traditionally, extended lymph node dissection (D2 dissection) has been considered as the standard gastric cancer surgery in Asia. However, the surgical treament of the INT-0116 trial was limited lymph node dissection (D0 or D1), therefore, this questioned the choice of gastrectomy in Asia. (4). It remains controversial whether adjuvant chemoradiotherapy can provide enhanced survival for patients with extensive lymph node dissection (D2). By contrast, in the United States and Europe, a lesser degree of gastric lymph node dissection (D1 dissection) has been widely preferred (6,7). To date, the extent of lymph node dissection for gastric cancer surgery has been a worldwide debate. Based on the positive data from several studies, such as the Dutch D1D2, Italian IGCSG-R01 and Taiwanese trials (8–10), D2 dissection surgery has been indicated to be an appropriate treatment option and has been incorporated into clinical practice in Western countries, resulting in lower morbidity and a higher five-year survival rate (11).

From these data, the aim of the present phase II trial was to evaluate the feasibility, efficacy and tolerability of adjuvant chemoradiotherapy following D2 gastrectomy on disease-free survival (DFS) and OS in 30 patients with stage II and IV (M0) gastric cancer.

## Patients and methods

### Patients

In total, 30 patients were recruited into the study according to the following eligibility criteria: Localized, histologically confirmed adenocarcinoma of the stomach, stages II and IV (M0) (according to the 2002 staging criteria of the American Joint Commission on Cancer); surgical resection of the tumor without residual disease (R0 gastrectomy); D2 lymph node dissection; and a caloric intake of >1,500 kcal per day (12). Patients with severe concurrent diseases, such as heart, renal or hepatic failure, secondary malignancies, chronic infections, neuropathy or diabetes were excluded. Furthermore, patients with a lack of comprehension of the protocol or the inability to comply with the requirements of the study were also ineligible.

This study was approved by the Medical Ethical Committee of The Affiliated Hospital of Binzhou Medical University (Shandong, China), and all patients submitted written informed consent.

### Surgery

The location of the primary tumor determined the type of surgical procedure. Frozen section confirmation of the negative margins was performed. The surgical requirement for eligibility was resection with curative intent and en bloc resection of the tumor, with negative margins. All patients underwent extensive (D2) lymph node dissection. This procedure involved the resection of all perigastric nodes and certain celiac, splenic or splenic-hilar nodes, hepatic arteries and cardiac lymph nodes, depending on the location of the tumor (13).

### Chemoradiotherapy

Therapy was administered on an out-patient basis. Subsequent to 2–4 weeks of surgical intervention, eligible patients were randomly scheduled to receive two cycles of adjuvant chemotherapy with a modified FOLFOX6 regimen. The treatment comprised a 2-h infusion of oxaliplatin (100 mg/m^2^) and leucovorin (100 mg/m^2^), followed by a 46-h continuous infusion of 5-fluorouracil (5-FU; 2,400 mg/m^2^) once every 3 weeks. Two cycles of chemotherapy were administered prior to and following chemoradiotherapy. The drug dose was reduced by 20% according to the non-hematological or hematological toxicity. Patients were excluded from the protocol treatment if distant metastasis developed. The clinical target volume (CTV) for radiotherapy was defined by pre-operative imaging. The CTV consisted of the gastric bed (with stomach remnant when present), anastomoses and draining lymph nodes, as described in the INT-0116 study (4). Multiple field three-dimensional conformation techniques (3D-CRT) and/or intensity modulated radiotherapy (IMRT) techniques were used in all patients. The patients underwent post-surgical chemoradiation of the marked area, and the total dose was 45 Gy, which consisted of 25 fractions of 1.8 Gy per day, for five days per week. Concurrent chemotherapy consisted of 350 mg/m^2^/day 5-FU via continuous infusion through a portable pump for five days each week (Saturdays and Sundays were excluded).

### Patient evaluation

Each patient was followed up every three months during the first year, then at six-month intervals for the next three years or until mortality. The evaluation consisted of a physical examination, laboratory tests, chest and abdominopelvic computed tomography (CT), gastroscopy and positron emission tomography/CT, if necessary. During the follow-up period, the suspected site and nodules were assessed using imaging studies, and even biopsy if possible. Relapse was classified as locoregional or distant. The locoregional relapse manifestations included surgical anastomosis, local regional recurrence associated with regional lymph node dissection, remnant stomach or gastric bed. The distant relapse manifestations included peritoneal seeding or metastasis of other organs. The site and the date of the first relapse and the date of mortality were recorded.

### Statistical analysis

Statistical calculations were performed using SPSS Statistics 16.0 for Windows (SPSS, Inc., Chicago, IL, USA). DFS and OS were defined as the intervals from the date of surgery to the date of relapse and mortality. The primary end-points of this study were feasibility and DFS. The second end-points were tolerability and OS. The median follow-up time was examined for all patients who survived. Follow-up time was defined as the date of the last visit for patients who survived. Patients who succumbed without documented disease recurrences were not considered for DFS analysis. However, these patients were included as mortalities for OS analysis. The Kaplan-Meier method was used to estimate the survival curves.

## Results

### Patient characteristics and treatment

A total of 30 patients (six female and 24 male) who underwent curative resection for gastric cancer with D2 lymph node dissection at the Affiliated Hospital of Binzhou Medical University were enrolled from the beginning of the study in January 2009. All patients were asymptomatic or ambulatory following gastrectomy. All patients received six cycles of adjuvant chemotherapy. All patients experienced the toxicities of the concurrent chemoradiotherapy. The patient characteristics and treatments are shown in Table I.

### Toxicity

Chemotherapy-related neutropenia, acute pancreatitis and other complications were observed. Toxicities were graded as 1 to 4 based on the National Cancer Institute Common Toxicity Criteria (14). The toxicities classified as grade 3 or higher are summarized in Table II. The most common toxicity was neutropenia. No patients succumbed to toxicity during the study. None of the patients had to be excluded from the study or could not undergo adjuvant chemoradiation.

### Survival and relapse

**Figure 1:**
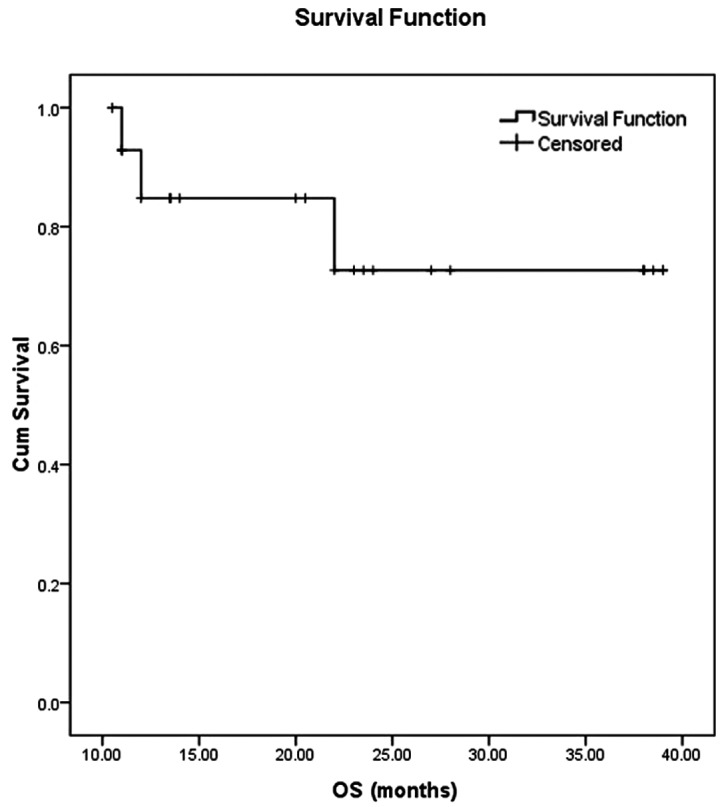
Kaplan-Meier survival curves for the overall survival (OS) of 30 patients with gastric cancer, showing a three-year OS rate of 72.7%.

**Figure 2:**
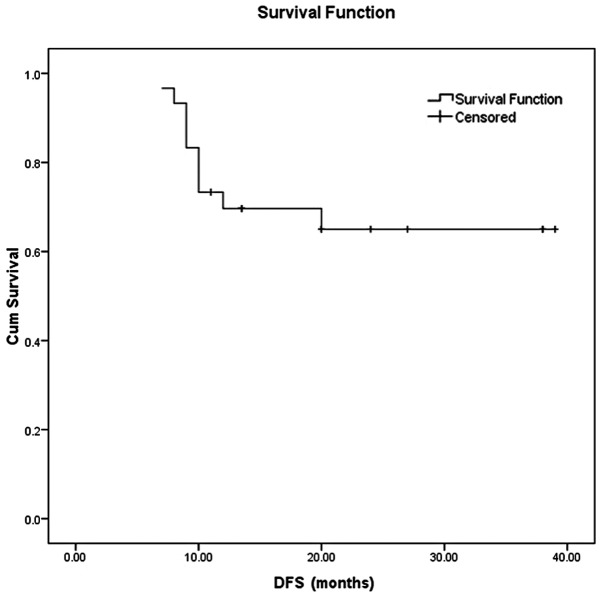
Kaplan-Meier survival curves for the disease-free survival (DFS) of 30 patients with gastric cancer, showing a three-year DFS rate of 65.0%.

The study was evaluated in October 31, 2013. At the time of analysis, relapse was observed in six patients. The median follow-up time was 21 months (range, 11–48 months) for patients with a disease-free status. The three-year OS and DFS rates were 72.7 and 65%, respectively (Figs. 1 and 2). Median OS and DFS were not determined, as 24 patients remained alive. A total of 10 patients relapsed during the follow-up period; of these patients, locoregional recurrence occurred in 2 (6.7%). Peritoneal relapse was reported in 4 (13.3%) patients, and another 4 (13.3%) patients suffered distant relapses (Table III).

## Discussion

East Asia has a high incidence rate of gastric cancer. Surgical resection is considered the most effective treatment for patients with gastric cancer. In total, 50–60% of patients with newly confirmed gastric cancer are suitable for radical resection, with modest five-year survival rates of 35–45% (15,16). Gastric cancer is not usually diagnosed immediately, resulting in the development of advanced-stage cancer in ~40% of patients with newly confirmed cancer (11). The advanced stage is characterized by a high degree of invasiveness and malignancy, and a low survival rate. The depth of tumor infiltration and lymph node involvement is closely associated with local recurrence and survival. This indicates that surgery may not prevent recurrence and distant metastasis. Auxiliary treatments, including radiotherapy, chemotherapy and chemoradiotherapy, have an important function. Recent studies have suggested that chemotherapy slightly improves survival times (17), similar to radiotherapy (18). No standard treatment for advanced gastric cancer is currently available. The INT-0116 study, which was a large prospective randomized study, clearly showed the benefit of chemoradiotherapy. A total of 36 and 54% of the patients in this study received D1 and D0 resections, respectively, while 9.7% received D2 resections. The problem with the adjuvant treatment was based on the adverse reactions of concurrent chemoradiotherapy in the study. Only 64% of patients completed the treatment as planned, and grade 3 and 4 adverse reactions were experienced by 41 and 32% of patients, respectively. Certain studies have indicated that the overall effect of this treatment is not ideal (11,19).

Compared with D1 resection, D2 radical resection has been shown to improve five-year OS (20), but does not increase the incidence and mortality from gastric cancer post-operative complications (8). The majority of Asian scholars have accepted that D2 is the standard surgical cure (21,22). With the 15-year follow-up results of a Dutch clinical study, Eastern and Western scholars reached a consensus for the first time to accept radical gastrectomy (D2) as the standard (8). In 2011, the National Comprehensive Cancer Network recommended that D2 resection be the standard surgery performed in experienced medical centers. Therefore, the number of randomized trials and non-randomized single institution studies increased, as a breakthrough in gastric cancer treatment was anticipated.

In the past years, patients have shown poor tolerance to conventional radiotherapy techniques, due to the toxicity to the liver, kidneys, intestines, spinal cord and other vital organs, as well as the inherent toxicity of radiotherapy. In novel 3D-CRT and IMRT multifield conformal irradiation techniques, dose distribution can be optimized on the target area, while radiation dose on the vital tissues surrounding the target and the organs affected can be reduced. In the present study, 3D-CRT techniques were used in all patients to avoid unnecessary toxicities. All eligible patients underwent the post-operative adjuvant chemoradiotherapy. The present study proposes that post-operative chemoradiotherapy following complete resection of gastric cancer with D2 lymphadenectomy, is feasible in a significant subset of patients. The present trial showed that the three-year OS and DFS rates were 72.7 and 65%, similar to the results found in other studies (23). Recently, the ARTIST trial has reported that chemoradiotherapy is not significantly beneficial in terms of OS (24). However, subgroup analysis of the ARTIST trial revealed that patients with lymph node metastasis had prolonged survival times with chemoradiotherapy.

Several studies have indicated the function of adjuvant radiotherapy with concurrent chemotherapy in gastric cancer in an extremely small portion of the patients who underwent the treatment (11,18). Results from the current study showed that post-operative chemoradiotherapy provided excellent locoregional control with acceptable and manageable treatment-related toxicity in patients with gastric cancer. However, as these findings were based on a small sampling set, validation with a larger cohort is required.

## Figures and Tables

**Table I:** Patient characteristics (n=30).

Characteristics	No. of patients	%
Age, years		
Median	51	
Range	34–65	
Gender		
Male	24	80.0
Female	6	20.0
Type of surgery		
Subtotal gastrectomy	22	73.3
Total gastrectomy	8	26.7
Stage		
II	8	26.7
III	18	60.0
IV	4	13.3
Lymph node		
Negative	2	6.7
Positive	28	93.3

**Table II:** Patients with hematological and non-hematological toxicities.

Type of toxic effect	No. of patients	%
Hematological		
Neutropenia	12	40.0
Anemia	0	0.0
Thrombocytopenia	0	0.0
Non-hematological		
Nausea	10	33.3
Vomiting	10	33.3
Diarrhea	2	6.7
Gastritis	0	0.0
Liver	1	3.3
Renal	0	0.0
Cutaneous	1	3.3
Sensory	7	23.3
Thromboembolic	0	0.0
Late toxicities	0	0.0

**Table III:** Pattern of treatment failure (n=10).

Site	No. of patients with relapse	%
Locoregional	2	6.7
Peritoneal	4	13.3
Distant	6	20.0

## Abbreviations

OS: overall survival
DFS: disease-free survival
3D-CRT: three-dimensional conformal radiotherapy
IMRT: intensity-modulated radiation therapy
CTV: clinical target volume
5-FU: 5-fluorouracil
CT: computed tomography
